# 

**Published:** 1907-07

**Authors:** 


**INDEX NUMBER.**

Sixty-second Year.

VOL. LXII. JULY, 1907. NO. 12

### BUFFALO MEDICAL JOURNAL

Established 1845 by AUSTIN FLINT M. D.

LIBRARY Indexed

### NOTICE OF REMOVAL

The Editorial Offices of this Journal have been removed to Delaware Court, 238 Delaware Avenue, where all communications, correspondence, exchanges and books for review should be addressed.

*William Warren Potter.*

